# Strength of Social Tie Predicts Cooperative Investment in a Human Social Network

**DOI:** 10.1371/journal.pone.0018338

**Published:** 2011-03-30

**Authors:** Freya Harrison, James Sciberras, Richard James

**Affiliations:** 1 Department of Zoology, University of Oxford, Oxford, United Kingdom; 2 Department of Biology and Biochemistry, University of Bath, Bath, United Kingdom; 3 Department of Physics, University of Bath, Bath, United Kingdom; Indiana University, United States of America

## Abstract

Social networks – diagrams which reflect the social structure of animal groups – are increasingly viewed as useful tools in behavioural ecology and evolutionary biology. Network structure may be especially relevant to the study of cooperation, because the action of mechanisms which affect the cost:benefit ratio of cooperating (e.g. reciprocity, punishment, image scoring) is likely to be mediated by the relative position of actor and recipient in the network. Social proximity could thus affect cooperation in a similar manner to biological relatedness. To test this hypothesis, we recruited members of a real-world social group and used a questionnaire to reveal their network. Participants were asked to endure physical discomfort in order to earn money for themselves and other group members, allowing us to explore relationships between willingness to suffer a cost on another's behalf and the relative social position of donor and recipient. Cost endured was positively correlated with the strength of the social tie between donor and recipient. Further, donors suffered greater costs when a relationship was reciprocated. Interestingly, participants regularly suffered greater discomfort for very close peers than for themselves. Our results provide new insight into the effect of social structure on the direct benefits of cooperation.

## Introduction

To a greater or lesser extent, most animal species live in groups for at least part of their lives and interactions between individuals affect the expression and evolution of behavioural traits [1], [2]. The social structure of a group of animals can be represented by a social network diagram which shows individuals as nodes connected by edges [3], [4], [5]. An edge connecting two individuals reflects the presence of some sort of social tie or interaction: grooming events, antagonistic encounters, physical proximity and sex are examples of interactions that may be used to construct networks (e.g. [6], [7], [8], [9], [10], [11]; see [3] for a review). Edges may simply reflect binary data (presence or absence of a ties, e.g. groomed or did not groom) or they may reflect continuous data that reflects the frequency or strength of the interaction (e.g. grooming frequency or duration). Knowing who interacts with whom (and how) adds a new facet to understanding population structure [12]. For example, network theory has been applied to studies of how diseases [13], [14] and behavioural memes [15], [16], [17] spread and evolve and to visualise group structuring based on kinship, [6], [10] age and sex [18] and behavioural type [19]. Network structure in its turn is likely to affect the expression and evolution of social traits and the value of applying a networks-based approach to animal – including human – behavioural ecology is increasingly recognised [3], [5], [11], [20], [21], [22], [23].

The evolution of cooperation is one question which could benefit from consideration within the framework of social networks. Alleles that cause their bearers to suffer some cost in order to increase another individual's direct fitness can be favoured if this behaviour results in increased inclusive fitness for the actor, due to direct (self) and/or indirect (kin-selected) fitness benefits [24], [25]. Direct benefits of cooperation arise when cooperative individuals can expect help in the future via direct or indirect reciprocity, when punishment or sanctions are imposed on non-cooperators or when cooperative individuals gain mating advantages (reviewed by [25], [26], [27]). The action of these mechanisms for maintaining cooperation can be enhanced when individuals obtain publicly-known reputations [28], for example due to image scoring [29], [30], [31] or gossip [32]. The nature of social ties between a pair of individuals in a group is likely to affect the operation of these mechanisms: network structure will affect the expected probability or frequency of repeated interactions and the flow of information between individuals. Therefore, social proximity may affect the expected benefits of cooperative interactions in much the same way as does biological relatedness [24], [33], [34], [35]. Further, characteristics of individuals – such as overall connectedness to other group members (degree), importance as a social “hub” linking other members (betweenness) and position in a social hierarchy - may influence the direct benefits of cooperating with that individual due to their ability to reciprocate, affect an actor's reputation, act as a conduit for generalised reciprocity, or impose sanctions. For instance, degree or betweenness of network members can vary considerably and may show a skewed distribution [36], [37], [38], [39], social policing may be carried out only by a small number of individuals in the group [40] and increased social rank may reflect increased resources for reciprocation, increased efficacy of reputation acquisition, increased ability to police or increased probability of aggression [41], [42], [43]. Additionally, it has been demonstrated in one primate species (stumptail macaque, *Macacca arctoides*), that low-ranking individuals may receive benefits from high-ranking individuals by associating with middle-ranking individuals [44]. This is consistent with the idea of the benefits of cooperation being passed on from one group member to another via their social network.

Computer simulations have shown that network structure can affect the relative fitness of cooperative genotypes [45], [46], [47], [48], [49] and a small number of experimental studies have explicitly shown the importance of social ties in determining whether individual animals engage in cooperative behaviours with other group members (e.g. predator inspection in guppies *Poecilia reticulata*, [50] and food sharing in spider monkeys *Ateles geoffroyi* [51]). With regard to our own species, one study [52] has explicitly explored cooperation in arbitrarily-constructed networks of humans. The authors showed that structuring groups of players into social networks increased the positive effect of generalised reciprocity on cooperation. In a similar experiment using groups of strangers, opportunities for social contact (communication) led to lower exploitation of a common resource pool [53]. A very small number of studies have tested for an effect of non-artificial social closeness on cooperation. A handful of economic studies have reported increased giving in various types of dictator game when the social distance between dictator and recipient is perceived to be smaller (e.g. [54], [55]). More recently, Haan et al [56] assigned high school students into groups comprised of classmates who either were or were not friends; they reported that contributions to a public good were higher when groups were composed of friends. Network thinking has also informed how we think about cooperation in networks of firms and thus how “social structure” of companies affects economic cooperation [57]. Given all this, it is surprising that no-one has yet attempted to test explicitly for a correlation between social distance and individual investment in cooperation in the context of a real human social network.

As an aside, we note that, as is the general case in studies of cooperation in humans, all of the studies listed above use a purely economic methodology, i.e. participants are given a sum of money for use in games. The ecological validity of this approach is not known, and a methodology where participants perform a time-consuming and physically demanding task in order to benefit one another [33] seems more in keeping with a desire to understand cooperation from the point of view of behavioural and evolutionary ecology.

We therefore wished to study cooperative interactions in the context of a revealed social network in order to address two hypotheses. Firstly, is willingness to cooperate with another individual correlated with the proximity of that individual in a social network? Secondly, what is the effect of reciprocated *versus* unreciprocated social relationships on cooperation? (i.e. what happens when A trusts B but B does not trust A?) We recruited members of a research unit and used a questionnaire to construct a weighted and directed network diagram which revealed the structure of the group. Members of the group then participated in an exercise where they endured physical discomfort in order to earn money for themselves and other group members, allowing us to look for relationships between willingness to suffer a cost on another person's behalf and the relative position of donor and recipient in the network. The effects of node-based characteristics such as seniority in the group hierarchy, social peer perception, degree and betweenness were also explored. We are not aware of any other study which takes this approach to investigate cooperation in a group of real organisms with a known network structure.

## Materials and Methods

### Ethics statement

Written informed consent was gained from all participants and this study was approved by the University of Oxford's Inter-divisional Research Ethics Committee for social science and humanities (ref. no. SSD/CUREC1/10 – 275).

### Recruitment of participants

We recruited 19 members (11 female) of a research unit at the University of Oxford; all participants were at least acquainted with one another. Participants were recruited by asking the head of the group to forward an invitation email to all of the group members; this was followed up by asking group members in person if they would be interested in participating. All group members who agreed to take part were recruited to the study. Participants included PhD students, post-doctoral researchers, fellows and administration staff. None of the participants were biological relatives and all except two had previously heard of game theory/the prisoner's dilemma.

### Building the network

Participants completed an online questionnaire about their relationships with and perceptions of each other. The questionnaire was designed and implemented using NetworkGenie [58] (https://secure.networkgenie.com). The full questionnaire consisted of four demographic questions (sex, familiarity with game theory, career stage and perceived relative position in the group seniority hierarchy) and fourteen network questions. Network questions provide response data in the form of matrices, containing either binary (yes/no, 1/0) or ordinal data (e.g. reflecting level of friendship or trust, frequency of interaction etc). The questionnaire and its reduction to a master matrix is detailed in Text S1. Briefly, questions which gave little information were discarded, some matrices which gave comparable information were combined and remaining matrices that were not strongly intercorrelated were summed to provide a master matrix. De Lange *et al*. [59] provide a detailed, worked example of how to develop a network questionnaire, though it should be noted that the exact type of questions asked and their informativeness is likely to depend on the nature of the group being considered. UCINET 6 [60] was used for all network analysis and construction and NetDraw [61] was used for network visualisation. The master matrix reflected level of friendship, level of perceived mutual trust, previous collaborative work and existence of strong positive or negative past interactions. Link weights were scaled such that they ranged from 0 to 1. We also calculated the betweenness of each node: the betweenness of the *i*th node is defined as the number of shortest paths between pairs of nodes other than *i* that pass through *i* (Freeman's standard measure of betweenness: [62]).

### Physical task

For each participant, we identified four recipients in the network. These were chosen to provide strong and weak in- and out-links in a cross-factored design. For each participant, we selected two of their strongest and two of their weakest out-links; one link in each pair had a corresponding in-link that differed from the out-link by less that 0.1 and the other had a corresponding in-link that differed from the out-link by ≥0.2. Full details of links between chosen pairs of participants are given in Text S1. We determined each participant's willingness to invest effort on behalf of their recipients using a physical task. 17 participants (8 female) were able to perform the physical task. Following the methodology of Madsen *et al*., [33], participants were asked to squat against the wall with their knees forming a 90° angle; this is a cross-country ski training exercise that quickly becomes painful. Participants were asked to squat for themselves and for their four recipients, with the five ‘rounds’ of effort in a randomised order. For every second spent squatting, participants earned £0.01 for the current recipient. There was no minimum or maximum time limit. The identity of recipients was provided on numbered cards, which participants turned over immediately before each ‘round.’ Participants were not told which of their peers would be squatting for them, were instructed not to discuss the experiment with one another and were informed that the task was not necessarily reciprocated; i.e. their recipients would not necessarily have the chance to earn money on their behalf. Participants began the squatting exercise simultaneously. The task was carried out in three-sided cubicles to prevent visual contact between participants and classical music was played during the task to prevent participants hearing one another's movements: these conditions were imposed to prevent individuals gaining information on each other's effort or attempting to compete with one another. Between each squatting period, participants were allowed to stretch and walk around to rest themselves until they felt ready to go on with the next recipient. Because the ease of this task depends on physical characteristics and fitness, the time spent squatting for each non-self recipient was standardised by dividing by the time the participant spent squatting for him or herself. A table of the standardised squatting times is provided in Text S1. At the end of the experiment, each participant was given a sum of money which corresponded to the total earned on their behalf by themselves and others. The average earning from the physical task was £6.75. The two participants who were unable to perform the physical task were each given a £10 shopping voucher.

### Analysis of data

Response matrices were analysed in UCINET [60] as detailed in Text S1; quadratic assignment procedure (QAP) was used to test for correlations between matrices and build the master matrix of in- and out-links. (See Text S1).

Network data should not be analysed using standard statistical tests based on ordinary least squares because of the inherent non-independence of dyadic interaction data: if one individual is an outlier, then all of the cells in the relevant row and/or column of a data matrix will also be outliers. Further, measures of node characteristics such as betweenness are not independent for the members of a network (because one agent's betweenness depends on that of the others). These problems and their solutions are discussed further by other authors [63], [64], [65], [66]. These pitfalls are avoided if a multiple matrix regression extension of the QAP procedure is used (MRQAP, as developed by Krackhardt [66] and Dekker *et al*. [63]; see also [67]). In short, MRQAP calculates partial matrix regression coefficients for a response matrix on several explanatory matrices and then uses a large number of random permutations of rows and columns within matrices to generate a sampling distribution and assign *p*-values.

We regressed the matrix of standardised time squatted on the explanatory matrices (master network matrix plus matrices of in- minus out-link, order, sex difference, hierarchy difference and recipient betweenness) using UCINET's MRQAP procedure with double Dekker semi-partialling and 10,000 permutations. Because our response matrix had many empty cells – each participant only squatted for four of the eighteen possible recipients – we required a way of ensuring these missing values did not influence the outcome of our analyses. Following the work of Cohen and Cohen [68] and Hemelrijk [65], we replaced all missing values in the matrices of standardised time squatted and squatting order with a value much greater than any of the non-missing values (10) and created a ‘dummy’ matrix variable which contained zeroes in cells for which response data was present and 10 in cells for which response data were missing. This dummy matrix was included in the analysis, allowing us to partial out the influence of missing data points and obtain estimates of partial correlation coefficients for our explanatory matrices.

## Results

**Figure 1:**
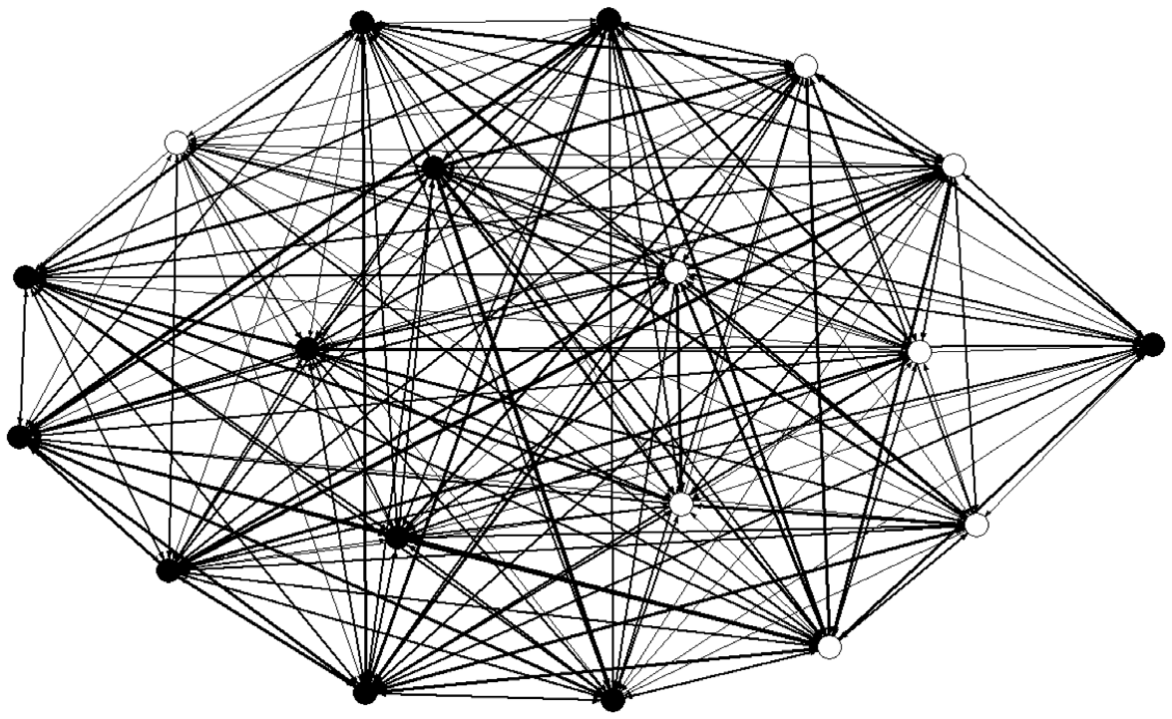
The master matrix (see Text S1) produced a directed and weighted network. Edge thickness reflects link weight (strength of relationship) and arrows show direction. Female participants are represented by closed circles, male participants by open circles.

**Figure 2:**
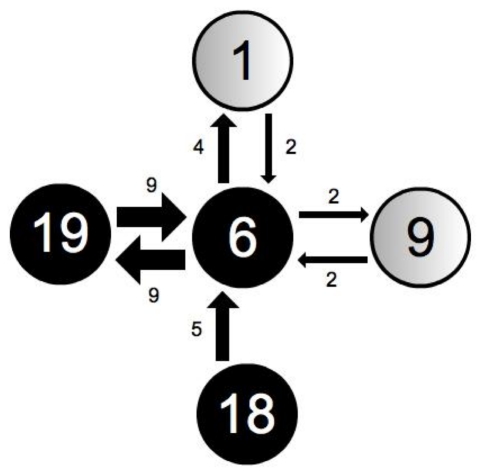
Links between one participant (number 6) and her chosen recipients. Participant number is given within the node and link weights shown numerically alongside links.

Figure 1 shows the social network of the group. The network consisted of one homogeneous component with no significant subgroup structuring, as determined by UCINET's *bi-component* algorithm: this measures whether any individuals act as ‘brokers’ between otherwise unconnected subgroups. Distributions of participant total in- and out-degree and betweenness were not significantly different from normal (Anderson-Darling tests, *p* = 0.614, 0.430 and 0.109, respectively. Further, the centralisation index based on Freeman's node-based measure of betweenness, which reflects the degree to which particular individuals may act as social ‘hubs’ was remarkably low at 0.27%. In a network where all nodes have the same betweenness, the centralisation index will be zero; its maximal value of 100% would result from a star graph. (For a useful comparison, see Wasserman & Faust's [69] exploration of marriage networks). These results suggest that this network shows little social substructure. Additionally, the matrix was fairly symmetric (QAP correlation of matrix vs. transposed matrix, *r* = 0.625, *p*<0.001; participant total in- and out-degrees were not significantly different: Wilcoxon *z* = 0.16, *p* = 0.872). However, some heterogeneity is evident in the peer-to-peer relationships of the group; these range from pairs of individuals who are connected only by virtue of belonging to this research group to pairs of very close friends. As an example of this heterogeneity, Figure 2 shows the links between participant 6 and her chosen recipients.

**Figure 3:**
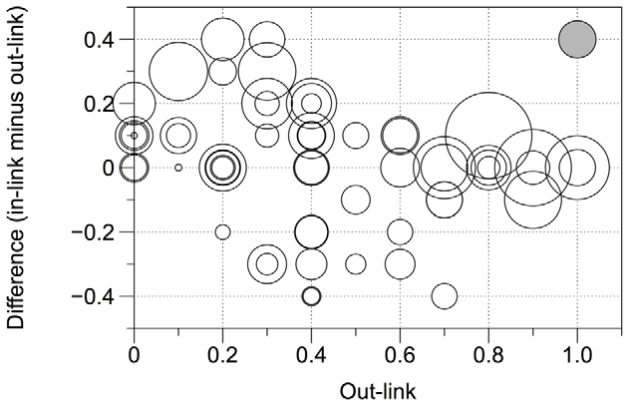
Effects of out-link strength and in- minus out-link on cooperative investment. Point size reflects standardised time squatted: white points show actual data, grey point is for reference only and corresponds to a standardised time of 1 Participants suffered a higher cost for recipients to whom they had declared a strong social link (*p<*0.001) and who had declared a a stronger social link to them (in-link minus out-link, *p* = 0.001).

We investigated the effects of total out- and in-link strength on standardised effort investment by regressing the matrix of standardised time squatted on explanatory matrices (master matrix plus matrices of in- minus out-link, order, sex difference, hierarchy difference and recipient betweenness) using MRQAP as described in the Materials & Methods. Non-significant terms were dropped from the full model in a stepwise manner to leave a minimal model, given in Table 1. We conclude that level of cooperative investment was positively correlated with out-link strength, in- minus out-link strength and order (*p*≤0.001). Thus people invested more effort for recipients to whom they reported a close social tie, and also for those recipients who reported a closer social tie to them. Figure 3 shows these results, and suggests that squatting time is higher when the in-link strength equals or exceeds the out-link strength, suggesting that participants are able to recognise when their peers place less value on a dyadic relationship than they do themselves, and correspondingly invest less effort on behalf of those peers. In other words, people invest more when social relationships are reciprocated. Participants tended to squat longer for recipients as the experimental session progressed (perhaps because they got used to the exercise, or because they initially wished to save energy for later squatting attempts). However, the regression coefficient for order was much smaller than those for the master matrix and of the in- minus out-link matrix (0.08 as compared with 0.26 and 0.20, respectively). Hierarchy difference, sex difference and recipient betweenness dropped out of the model (p = 0.10, 0.41 and 0.21, respectively).

**Table 1:** Results of MRQAP using a) master matrix and b) components of master matrix.

*a)*		
independent variable	regression coefficient	*p*
intercept	0.60	-
missing values	0.85	<0.001
master matrix	0.26	<0.001
in- minus out-link	0.20	0.001
order	0.08	<0.001

*b)*
independent variable	regression coefficient	*p*
intercept	0.63	-
missing values	0.86	<0.001
order	0.08	<0.001
friendship strength	0.10	<0.001
past collaboration	0.05	0.027

See main text for explanation of MRQAP. Unstandardised regression coefficients are shown and *p*-values are based on 10,000 random permutations of matrices. R^2^_adj_ = 0.998, combined *p*<0.001 for both models.

We also wished to explore the effects of the individual components of the master matrix. Therefore we conducted a further MRQAP analysis, beginning with a full model that included order, sex difference, hierarchy difference, recipient betweenness and the four components of the master matrix: friendship strength, perceived level of mutual trust, incidence of past collaboration and strong past experiences. The only significant predictors of standardised time squatted were order, friendship strength and incidence of past collaboration. (*p*≤0.027; see Table 1b). All regression coefficients were small compared with those obtained in the analysis of the master matrix.

It is notable that the standardised time squatted often exceeded 1; the tendency for participants to squat longer for very close recipients than for themselves is evident in Figure 3. In fact, 10/17 participants recorded a standardised squatting time of >1 for at least one recipient; one participant recorded a standardised time of >1 for all recipients.

## Discussion

We present the first (to our knowledge) explicit analysis of relative cooperative investment in a real-world human social network. We have used an experimental method with high ecological validity (recruitment of a real social group and use of a task which incurs a recognisable physical cost) to provide new insight into the effect of social structure on the likely direct benefits of cooperation for different individuals in a group. We report that social proximity increased cooperative investment in a manner analogous to biological relatedness [24], [33], [34], [35]. This finding is consistent with analogous studies linking cooperation and social closeness in two other species. One of us has previously reported [50] that female guppies disproportionately engage in cooperative predator inspection with others with whom they have a strong social association in the wild, and the incidence of food sharing in spider monkeys has been shown to be correlated with incidence of affiliative behaviour (allogrooming; [51]). Our results are also consistent with published data on the behaviour of friends and non-friends in public goods games [56]. Additionally, we show that participants invested more in peers who placed equal or greater value on the dyadic relationship, compared with their own assessment of the relationship.

That social proximity appears to function in an analogous manner to biological relatedness is in some ways unsurprising. Queller [70] has shown that the mathematics of inclusive fitness theory and reciprocity are essentially identical – which should be self evident, because both are founded on the existence of a positive covariance between an actor's expression of a cooperative behaviour and the probability of the recipient carrying alleles that promote the same behaviour. Therefore, if the likelihood of individuals reciprocating cooperative acts is positively correlated with their social proximity to the actor, we expect the exactly the pattern of behaviour found in our experiment. Further, a reliance on reciprocity is consistent with our conclusion that participants invested more when the recipient reciprocated or over-reciprocated their perceived social relationship.

It is easy to see how social proximity could increase opportunities for reciprocity, as a simple function of frequency or duration of interactions. Further, there is some evidence that individuals with cooperative phenotypes tend to cluster together in networks (in network parlance, the tendency for neighbours to share phenotypes is termed assortment or homophily). In a recent publication, Brañas-Garza et al [71] note that individuals who offer a larger share of the pie in a dictator game are more socially integrated (as measured by betweenness and number of bidirectional edges). In another dictator game played in a real social group, Lieder et al [55] report that people who display relatively high levels of altruism tend to have friends who also display relatively high levels of altruism. Such assortment of cooperative phenotypes would increase the gradient of the expected relationship between social proximity and probability of reciprocation. In fact, it has long been argued in one way or another that assortment, such that cooperative individuals interact with one another more frequently than with non-cooperators, increases the relative fitness of cooperator genotypes (e.g. [48], [72], [73]. Unfortunately, given the homogeneity of our network, we cannot really explore the assortment hypothesis in this case.

A particularly interesting observation from our study is the participants' willingness to invest more effort on the behalf of some of their peers (those with whom they share a very strong social tie) than for themselves. This contrasts with Madsen *et al*'s study [33], which used the same methodology and in which participants did not invest more effort for any class of relative than they did for themselves. One explanation for this discrepancy could be increased importance of or potential for direct benefits stemming from reciprocity or reputation in a social as opposed to family setting. On the simplest level, one could imagine that individuals might expect relatives to help them regardless of their own past behaviour if the indirect (kin-selected) benefits of helping are sufficiently high [24]; in the absence of significant indirect fitness ties between non-kin more effort must be expended in helping in order to ensure reciprocal conferment of help in the future. This is an area that would benefit from explicit empirical and theoretical exploration.

This particular network was well-connected and homogeneous, characteristics that potentially explain the lack of influence of variables other than tie strength, such as any “extra” benefits of cooperation that could stem from the recipient's social position. Further, levels of declared friendship were fairly high in this network. A more diffuse or subdivided network, with skewed betweenness and/or degree distributions and perhaps lower levels of friendship between its members, would provide a better test for potential effects of trust, past experience, betweenness and hierarchy. It would also allow us to explore the hypothesis that cooperative individuals tend to cluster together. Such networks could potentially be found in large companies, where individuals do not work together so closely as is common in research units. Local neighbourhoods in urban communities may be useful for similar reasons.

We present in this work a methodology which is relatively novel and which, we argue, contains aspects which have been under-utilised in studies of cooperative behaviour. We would therefore like to discuss three specific areas which would benefit from further thought and development. Firstly, we picked one of several potential ways to standardise squat time. As the originators of this measure of investment effort [33] had standardised by time squatted for oneself, we also used this method. However, it may be argued that the small but significant effect of order on relative squat time could render this approach problematic. One alternative would be to first ask participants to squat until they reach a self-reported pain threshold, and standardise all times by this threshold. Any effect of order (due perhaps to acclimatisation to the exercise) might then more neatly partial out of the model and give a more reliable estimate of the effect sizes of the variables under investigation. However, because it is not clear how to numerically represent ‘self’ in our independent matrices, this approach would be problematic if we specifically wanted to investigate investment in others *versus* self.

Secondly, it should be noted that the network in our study was not complete, in that not all members of the research unit took part in the study. This may affect node-based measures such as betweenness, may imply that we captured only a single component of a multi-component network and/or mean that we are missing individuals from the periphery of the social group [21]. However, we did uncover a range of link strengths, from pairs of individuals that were connected only by virtue of belonging to this group to pairs of individuals who were very close friends, so while our power may have been low it is unlikely that our results are invalidated by incomplete sampling. We must also acknowledge the incompleteness of our dependent matrix (time invested) – for obvious considerations of time and participant fatigue, we had to limit the number of recipients for whom each participant squatted. While we have a statistically sound method for dealing with the ensuing missing variables, the signal from these values may swamp that from our explanatory variables and we may have underestimated the strength of the effect of social proximity.

Thirdly, we are well aware of the potential pitfalls [74] of the stepwise regression approach taken in our analysis. However, robust criteria for model comparison such as AIC or BIC are not available to us when we use MRQAP – which is demanded by the intrinsic non-independence of network data. MRQAP does provide us with measures of R^2^, which can be used as a criterion for comparing nested models, but the huge signal from the missing values matrix discussed above inflated R^2^ to the point where it was essentially the same for all models. If a network approach is to be more widely used in behavioural ecology – and we strongly believe that it has much to offer – more theoretical work on the statistical analysis of network data, particularly from the perspective of model choice, is essential.

In summary, we present our results as novel and preliminary observations that support further and more complete exploration of cooperation in real-world social groups. A study of a sample of different social networks could usefully test whether the old adage that one may choose one's friends, but not one's relatives, has a bearing on social investment rules.

## Supporting Information

Text S1 Development of network questionnaire and raw task data.
(DOC)

## References

[1] Krause J, Ruxton G (2002). Living in Groups..

[2] Krause J, Lusseau D, James R (2009). Animal social networks: an introduction.. Behav Ecol Sociobiol.

[3] Croft D, James R, Krause J (2008). Exploring Animal Social Networks..

[4] Newman MEJ (2003). The structure and function of complex networks.. SIAM Review.

[5] Scott J (2000). Social Network Analysis..

[6] Gero S, Engelhaupt D, Whitehead H (2008). Heterogeneous social associations within a sperm whale, *Physeter macrocephalus*, unit reflect pairwise relatedness.. Behav Ecol Sociobiol.

[7] Lusseau D (2003). The emergent properties of a dolphin social network.. Proc Biol Sci.

[8] Pike TW, Samanta M, Lindstrøm J, Royle NJ (2008). Behavioural phenotype affects social interactions in an animal network.. Proc Biol Sci.

[9] Voelkl B, Kasper C (2009). Social structure of primate interaction networks facilitates the emergence of cooperation.. Biol Lett.

[10] Wolf JBW, Trillmich F (2008). Kin in space: social viscosity in a spatially and genetically substructured network.. Proc Biol Sci.

[11] Madden J, Drewe J, Pearce G, Clutton-Brock T (2009). The social network structure of a wild meerkat population: 2. Intragroup interactions.. Behav Ecol Sociobiol.

[12] Newman MEJ, Girvan M (2004). Finding and evaluating community structure in networks.. Phys Rev E.

[13] Gupta S, Anderson RM, May RM (1989). Networks of sexual contacts - implications for the pattern and spread of HIV.. Aids.

[14] Eames KTD, Read JM, Edmunds WJ (2009). Epidemic prediction and control in weighted networks.. Epidemics.

[15] Franks DW, Noble J, Kaufman P, Stagi S (2008). Extremism propagation in social networks with hubs.. Adaptive Behavior.

[16] Fowler JH, Christakis NA (2010). Cooperative behavior cascades in human social networks.. PNAS.

[17] Centola D (2010). The spread of behavior in an online social network experiment.. Science.

[18] Wolf JBW, Mawdsley D, Trillmich F, James R (2007). Social structure in a colonial mammal: unravelling hidden structural layers and their foundations by network analysis.. Anim Behav.

[19] Croft D, Krause J, Darden S, Ramnarine I, Faria J (2009). Behavioural trait assortment in a social network: patterns and implications.. Behav Ecol Sociobiol.

[20] Dunbar RIM (2008). Cognitive constraints on the structure and dynamics of social networks.. Group Dynamics: Theory, Research and Practice.

[21] James R, Croft D, Krause J (2009). Potential banana skins in animal social network analysis.. Behav Ecol Sociobiol.

[22] Proulx SR, Promislow DEL, Phillips PC (2005). Network thinking in ecology and evolution.. Trends Ecol Evol.

[23] Sih A, Hanser S, Mchugh K (2009). Social network theory: new insights and issues for behavioral ecologists.. Behav Ecol Sociobiol.

[24] Hamilton WD (1964). The genetical evolution of social behaviour I & II.. J Theor Biol.

[25] Gardner A, Foster KR, Korb J, Heinze J (2008). The Evolution and Ecology of Cooperation - History and Concepts.. Ecology of Social Evolution.

[26] Frank SA (1998). Foundations of social evolution..

[27] Hammerstein P, Leimar O (2006). Cooperating for direct fitness benefits.. J Evol Biol.

[28] Milinski M, Semmann D, Krambeck H-J (2002). Reputation helps solve the ‘tragedy of the commons’.. Nature.

[29] Nowak MA, Sigmund K (1998). Evolution of indirect reciprocity by image scoring.. Nature.

[30] Milinski M, Semmann D, Bakker TCM, Krambeck HJ (2001). Cooperation through indirect reciprocity: image scoring or standing strategy?. Proc Biol Sci.

[31] Wedekind C, Milinski M (2000). Cooperation through image scoring in humans.. Science.

[32] Sommerfeld RD, Krambeck H-J, Semmann D, Milinski M (2008). Gossip as an alternative for direct observation in games of indirect reciprocity.. PNAS.

[33] Madsen EA, Tunney RJ, Fieldman G, Plotkin HC, Dunbar RI (2007). Kinship and altruism: a cross-cultural experimental study.. Brit J Psychol.

[34] Ruch J, Heinrich L, Bilde T, Schneider J (2009). Relatedness facilitates cooperation in the subsocial spider, *Stegodyphus tentoriicola*.. BMC Evol Biol.

[35] Russell AF, Hatchwell BJ (2001). Experimental evidence for kin-biased helping in a cooperatively breeding vertebrate.. Proc Biol Sci.

[36] Csányi G, Szendröi B (2004). Structure of a large social network.. Phys Rev E.

[37] Fowler JH, Dawes CT, Christakis NA (2009). Model of genetic variation in human social networks.. PNAS.

[38] Lusseau D, Newman MEJ (2004). Identifying the role that animals play in their social networks.. Proc Biol Sci.

[39] McDonald DB (2007). Predicting fate from early connectivity in a social network.. PNAS.

[40] Flack JC, Girvan M, de Waal FBM, Krakauer DC (2006). Policing stabilizes construction of social niches in primates.. Nature.

[41] Seyfarth RM (1976). Social relationships among female baboons.. Anim Behav.

[42] Seyfarth RM (1980). The distribution of grooming and related bejaviours among adult female vervet monkeys.. Anim Behav.

[43] Drea CM, Carter AN (2009). Cooperative problem solving in a social carnivore.. Anim Behav.

[44] Estrada A, Sandoval JM, Manzolillo D (1978). Further data on predation by free-ranging stumptail macaques *Macacca arctoide*s.. Primates.

[45] Tomochi M (2004). Defectors' niches: prisoner's dilemma game on disordered networks.. Social Networks.

[46] Ohtsuki H, Hauert C, Lieberman E, Nowak MA (2006). A simple rule for the evolution of cooperation on graphs and social networks.. Nature.

[47] Lehmann L, Keller L, Sumpter DJT (2007). The evolution of helping and harming on graphs: the return of the inclusive fitness effect.. J Evol Biol.

[48] Santos FC, Rodrigues JF, Pacheco JM (2006). Graph topology plays a determinant role in the evolution of cooperation.. Proc Biol Sci.

[49] Santos FC, Santos MD, Pacheco JM (2008). Social diversity promotes the emergence of cooperation in public goods games.. Nature.

[50] Croft D, James R, Thomas P, Hathaway C, Mawdsley D (2006). Social structure and co-operative interactions in a wild population of guppies (*Poecilia reticulata*).. Behav Ecol Sociobiol.

[51] Pastor-Nieto R (2001). Grooming, kinship, and co-feeding in captive spider monkeys *(Ateles geoffroyi*).. Zoo Biology.

[52] Yamagishi T, Cook KS (1993). Generalized exchange and social dilemmas.. Soc Psychol Quart.

[53] Cardenas JC, Stranlund J, Willis C (2000). Local environmental control and institutional crowding-out.. World Development.

[54] Jones B, Rachlin H (2006). Social discounting.. Psychol Sci.

[55] Leider S, Möbius MM, Rosenblat T, Do Q-A (2009). Directed altruism and enforced reciprocity in social networks.. Quart J Econ.

[56] Haan M, Kooreman P, Riemersma T (2006). Friendship in a Public Good Experiment.. IZL Discussion Paper series No. 2108.

[57] Håkansson H (1989). Corporate technological behaviour: co-operation and networks: Routledge.

[58] Hansen WB, Reese E, Bryant KS, Bishop D, Wyrick CH Network Genie..

[59] De Lange D, Agneessens F, Waege H (2004). Asking social network questions: a quality assessment of different measures.. Metodoloski zvevki.

[60] Borgatti SP, Everett MG, Freeman LC (2002). Ucinet for Windows: software for social network analysis..

[61] Borgatti SP (2002). NetDraw network visualization..

[62] Freeman LC (1979). Centrality in social networks: conceptual clarification.. Social Networks.

[63] Dekker D, Krackhardt D, Snijders TAB (2007). Sensitivity of MRQAP tests to collinearity and autocorrelation conditions.. Psychometrika.

[64] Hemelrijk CK (1990). Models of, and tests for, reciprocity, unidirectionality and other social interaction patterns at a group level.. Anim Behav.

[65] Hemelrijk CK (1990). A matrix partial correlation test used in investigations of reciprocity and other social interaction patterns at group level.. J Theor Biol.

[66] Krackhardt D (1988). Predicting with networks - nonparametric multiple-regression analysis of dyadic data.. Social Networks.

[67] Gibbons D, Olk PM (2003). Individual and structural origins of friendship and social position among professionals.. J Pers Soc Psychol.

[68] Cohen J, Cohen P (1983). Applied multiple regression/correlation analysis for the behavioural sciences..

[69] Wasserman S, Faust K (1994). Social network analysis: methods and applications..

[70] Queller DC (1985). Kinship, reciprocity and synergism in the evolution of social behaviour.. Nature.

[71] Brañas-Garza P, Cobo-Reyes R, Espinosa MP, Jiménez N, Kovárík J, Ponti G (2010). Altruism and social integration.. Games Econ Behav.

[72] Axelrod R (1984). The Evolution of Cooperation..

[73] Hruschka DJ, Henrich J (2006). Friendship, cliquishness, and the emergence of cooperation.. J Theor Biol.

[74] Whittingham MJ, Stephens PA, Bradbury RB, Freckleton RP (2006). Why do we still use stepwise modelling in ecology and behaviour?. J Anim Ecol.

