# Supplementary material for: Strength of Social Tie Predicts Cooperative Investment in a Human Social Network
Source: PLoS One. 2011 Mar 30;6(3):e18338. doi: 10.1371/journal.pone.0018338 (PMC3068164; doi:10.1371/journal.pone.0018338)
Supplement: Text S1 — Development of network questionnaire and raw task data. (DOC) [file pone.0018338.s001.doc]

**Strength of social tie predicts cooperative investment in a human social network.**

***Text S1: development of network questionnaire and raw task data***

**Freya Harrison^1,2^*, James Sciberras^1^ & Richard James^3^**

^1^ Department of Zoology, University of Oxford, South Parks Road, Oxford OX1 3PS UK

^2^ Department of Biology & Biochemistry, University of Bath, Claverton Down, Bath BA2 7AY, UK

^3^ Department of Physics, University of Bath, Claverton Down, Bath BA2 7AY, UK

* Author for correspondence: freya.andersdottir@gmail.com

**A) Full questionnaire answered by participants.**

| **Person-centred (demographic) questions** | | |
| --- | --- | --- |
| A | Are you Male or Female?  a) Male  b) Female | |
| B | Have you ever heard of Game Theory or the Prisoner's Dilemma?  a) No  b) Yes | |
| C | At what level in your academic career are you?  a) Graduate Student  b) Research Assistant  c) Post-doc  d) Fellow or Lecturer  e) Non-academic (administration) staff | |
| D | Please select at what stage in their academic career the other people in [name of research group] are compared to you.  a) Senior to me  b) The same as me  c) Junior to me | |
| **Network questions** | | **Value to matrix** |
| 1 | Are you related to any of the people listed below? *[All group members listed, participant selects]* | 1 if selected |
| 2 | Who do you spend time with outside of [name of research group] (for example, in free time)? *[All group members listed, participant selects]* | 1 if selected |
| 3 | Which of these people are your friends? (A friend is someone you like and who you actively choose to spend time with). *[All group members listed, participant selects]* | 1 if selected |
| 4 | People play many roles. Which role best describes your relationship with the people listed below? (If you are related to any of these people, please ignore this fact for the moment). *[Group members nominated in Q3 listed, participant assigns category]*  a) Friends - we get on well  b) Good friends - we spend a lot of time together  c) Very close friends  d) This person is my boyfriend, girlfriend or partner | 1  2  3  4 |
| 5 | Do you and any of the following people belong to the same club or society (outside of work, such as a sports team etc)? *[All group members listed, participant selects]* | 1 if selected |
| 6 | Do you live with any of the following people? *[All group members listed, participant selects]* | 1 if selected |
| 7 | Do you actively dislike anyone listed below? *[All group members listed, participant assigns category]*  a) Yes, I dislike this person  b) No, I do not dislike this person  c) Do not know / no opinion | -1  0  0 |
| 8 | How much do you trust the people listed below? Note: An average person is someone you do not know, i.e. a random person in the street. *[All group members listed, participant assigns category]*  a) Less than an average person  b) The same as an average person  c) More than an average person  d) Do not know / no opinion | -1  0  1  0 |
| 9 | How much do you believe the people listed below trust you? Note: An average person is someone you do not know, i.e. a random person in the street. *[All group members listed, participant assigns category]*  a) Less than an average person  b) The same as an average person  c) More than an average person  d) Do not know / no opinion | -1  0  1  0 |
| 10 | If you received some personal bad news (for example, a family tragedy) and you had to tell one person in [name of research group] at your next meeting, who would it be? *[All group members listed, participant selects]* | 1 if selected |
| 11 | Have you had a strong positive past experience (such as intimacy, good working relationship etc) with any of the people listed below? *[All group members listed, participant assigns category]*  a) Yes  b) No | 1  0 |
| 12 | Have you had a strong negative past experience (such as a fight, bad working relationship etc) with any of the people listed below? *[All group members listed, participant assigns category]*  a) Yes  b) No | -1  0 |
| 13 | Have you ever had any scientific collaboration (includes giving advice through to writing a paper together) with any of the people listed below. *[All group members listed, participant selects]* | 1 if selected |
| 14 | How often have you or do you collaborate with the individuals you have selected? *[Group members nominated in Q13 listed, participant assigns category]*  a) Once  b) A few times  c) Frequently  d) Almost all the time | 1  2  3  4 |

**B) Reducing network items to produce master matrix**

Response matrices were combined to form a master network matrix in a two-step procedure. In the first step, questions were discarded or combined based on information content:

| **Question** | **Decision** | **Notes** |
| --- | --- | --- |
| 1 | Discard | No participants were related. |
| 2 | Retain |  |
| 3 | Discard | This question was simply a filter for question 4. |
| 4 | Retain |  |
| 5 | Discard | Very asymmetric, which would not be expected – question was probably interpreted differently by different participants. |
| 6 | Discard | Only one pair of participants lived together and they were the only couple in the group, so unlikely to provide any extra information. |
| 7 | Discard | Only three instances of dislike and these were also picked up on in Q4, 8 and 12. |
| 8 | Retain | Combine with Q9 |
| 9 | Retain | Combine with Q8 |
| 10 | Retain |  |
| 11 | Retain | Combine with Q12 |
| 12 | Retain | Combine with Q11 |
| 13 | Retain |  |
| 14 | Discard | Very asymmetric, which would not be expected – question was probably interpreted differently by different participants. |

Questions 11 and 12 were summed to provide a single matrix reflecting strong past experience. The matrices corresponding to questions 8 and 9 were summed in order to provide a measure of the perceived level of mutual trust.

This step therefore resulted in six matrices remaining to be considered. We tested whether there were significant intercorrelations between pairs of these matrices using the quadratic assignment procedure implemented in UCINET 6 (Borgatti, S. P., Everett, M. G. & Freeman, L. C. 2002, Ucinet for Windows: software for social network analysis. Harvard, MA: Analytic Technologies) with 10,000 random permutations. Correlation coefficients are shown below:

|  | Q2 | Q4 | Q8+Q9 | Q10 | Q11+Q12 | Q13 |
| --- | --- | --- | --- | --- | --- | --- |
| Q2 (Time) | *1.0* |  |  |  |  |  |
| Q4 (Friendship roles) | .62 | *1.0* |  |  |  |  |
| Q8+Q9 (Trust) | .32 | .33 | *1.0* |  |  |  |
| Q10 (Bad news) | .27 | .52 | .25 | *1.0* |  |  |
| Q11+Q12 (Strong past experience) | .32 | .37 | .38 | .25 | *1.0* |  |
| Q13 (Collaboration) | .42 | .51 | .29 | .26 | .28 | *1.0* |

All six matrices were intercorrelated (*p* < 0.001), but most of the correlation coefficients were low. Questions whose matrices were not strongly correlated were assumed to provide different types of data on social ties and were retained. Two questions (bad news and time) were strongly correlated with the “friendship roles” matrix and so were discarded. The question on collaboration also had a fairly strong correlation with the “friendship roles”matrix but given that it reflects one of the primary professional ties between academic researchers we decided to retain it. The remaining four matrices were summed and the sum divided by 10 (the maximum value) to create a master matrix with link weights ranging from 0 to 1, shown below with rows representing out-links:

|  | **1** | **2** | **3** | **4** | **5** | **6** | **7** | **8** | **9** | **10** | **11** | **12** | **13** | **14** | **15** | **16** | **17** | **18** | **19** |
| --- | --- | --- | --- | --- | --- | --- | --- | --- | --- | --- | --- | --- | --- | --- | --- | --- | --- | --- | --- |
| **1** |  | 0.4 | 0.3 | 0.3 | 0.2 | 0.2 | 0.9 | 0.3 | 0.3 | 0.1 | 0.3 | 0.5 | 0.3 | 0.5 | 0.4 | 0.2 | 0.6 | 0.3 | 0.3 |
| **2** | 0.6 |  | 0.7 | 0.2 | 0.4 | 0.5 | 0.3 | 0.4 | 0.4 | 0.4 | 0.2 | 0.3 | 0.6 | 0.5 | 0.4 | 0.2 | 0.3 | 0.8 | 0.4 |
| **3** | 0.6 | 0.6 |  | 0.4 | 0.2 | 0.3 | 0.3 | 0.2 | 0.4 | 0 | 0.4 | 0 | 0 | 0.2 | 1 | 0.2 | 0 | 0.2 | 0.4 |
| **4** | 0.5 | 0.6 | 0.7 |  | 0.4 | 0.4 | 0.2 | 0.6 | 0.7 | 0.2 | 0.9 | 0.4 | 0.2 | 0.2 | 0.2 | 0.2 | 0.5 | 0.2 | 0.5 |
| **5** | 0.4 | 0.4 | 0.4 | 0.4 |  | 0.4 | 0.4 | 0.4 | 0 | 0.4 | 0.4 | 0.6 | 0.4 | 0.4 | 0.4 | 0 | 0 | 0.7 | 0.4 |
| **6** | 0.4 | 0.4 | 0.6 | 0.2 | 0.4 |  | 0.4 | 0 | 0.2 | 0.3 | 0.2 | 0 | 0 | 0.4 | 0.4 | 0.2 | 0.2 | 0 | 0.9 |
| **7** | 0.8 | 0.2 | 0.4 | 0 | 0.2 | 0.3 |  | 0.2 | 0.2 | 0.2 | 0 | 0.6 | 0 | 0.4 | 0.3 | 0 | 0 | 0.2 | 0.2 |
| **8** | 0.6 | 0.7 | 0.5 | 0.8 | 0.3 | 0.4 | 0.2 |  | 0.2 | 0.7 | 0.7 | 0.3 | 0.6 | 0.5 | 0.5 | 0.2 | 0.3 | 0.3 | 0.3 |
| **9** | 0.4 | 0.3 | 0.5 | 0.1 | 0.1 | 0.2 | 0.3 | 0.1 |  | 0.1 | 0.2 | 0.3 | 0.2 | 0.3 | 0.4 | 0.2 | 0.1 | 0.4 | 0.2 |
| **10** | 0.2 | 0.4 | 0.3 | 0 | 0.6 | 0.3 | 0.3 | 0.6 | 0 |  | 0.5 | 0.2 | 0.6 | 0.4 | 0.3 | 0 | 0.2 | 0 | 0.7 |
| **11** | 0.6 | 0.4 | 0.5 | 0.7 | 0.4 | 0.2 | 0.4 | 0.4 | 0.4 | 0.5 |  | 0.5 | 0.5 | 0.2 | 0.5 | 0.1 | 0.4 | 0.4 | 0.4 |
| **12** | 0.6 | 0.4 | 0.1 | 0.4 | 0.7 | 0.4 | 0.4 | 0.5 | 0.3 | 0 | 0.2 |  | 0.3 | 0.3 | 0.4 | 0 | 0.4 | 0.7 | 0.3 |
| **13** | 0.5 | 0.7 | 0.2 | 0.5 | 0.3 | 0.2 | 0.3 | 0.4 | 0.2 | 0.4 | 0.3 | 0.3 |  | 0.2 | 0.2 | 0.2 | 0.2 | 0.5 | 0.6 |
| **14** | 0.4 | 0.3 | 0.3 | 0.3 | 0.2 | 0.2 | 0.4 | 0.2 | 0.2 | 0.2 | 0.2 | 0.3 | 0.2 |  | 0.3 | 0.2 | 0.3 | 0.2 | 0.2 |
| **15** | 0.6 | 0.6 | 1 | 0.4 | 0.3 | 0.4 | 0.3 | 0.4 | 0.6 | 0.2 | 0.6 | 0.5 | 0.2 | 0.4 |  | 0.2 | 0 | 0.2 | 0.7 |
| **16** | 0.4 | 0.2 | 0.2 | 0.4 | 0.1 | 0.1 | 0.1 | 0.4 | 0.1 | 0.1 | 0.1 | 0.2 | 0.1 | 0.4 | 0.1 |  | 0.1 | 0.1 | 0.1 |
| **17** | 0.6 | 0 | 0 | 0.2 | 0 | 0 | 0.1 | 0.1 | 0 | 0.1 | 0.2 | 0.6 | 0.1 | 0.5 | 0.1 | 0.1 |  | 0.1 | 0.2 |
| **18** | 0.5 | 0.8 | 0.3 | 0.2 | 0.7 | 0.5 | 0.4 | 0.4 | 0.5 | 0.2 | 0.3 | 0.7 | 0.4 | 0.2 | 0.2 | 0.2 | 0.2 |  | 0.5 |
| **19** | 0.6 | 0.5 | 0.6 | 0.4 | 0.4 | 0.9 | 0.4 | 0.4 | 0.4 | 0.6 | 0.4 | 0.6 | 0.5 | 0.5 | 0.7 | 0.2 | 0.4 | 0.5 |  |

Our methodology for developing this questionnaire is based on the more detailed questionnaire developed and explored by De Lange, D, Agneessens, F. & Waege, H. 2004, *Metodoloski Zvezki* **1**:351-378.

**C) Raw data for physical task (non-self recipients only)**

| **Donor** | **Recipient** | **In** | **Out** | **Diff** | **StTime** | **Order** |  | **Donor** | **Recipient** | **In** | **Out** | **Diff** | **StTime** | **Order** |
| --- | --- | --- | --- | --- | --- | --- | --- | --- | --- | --- | --- | --- | --- | --- |
| 1 | 7 | 0.8 | 0.9 | -0.1 | 1.53 | 5 |  | 9 | 12 | 0.2 | 0.4 | -0.2 | 0.87 | 4 |
| 1 | 10 | 0.2 | 0.1 | 0.1 | 0.65 | 1 |  | 9 | 13 | 0.2 | 0.2 | 0 | 0.86 | 3 |
| 1 | 14 | 0.4 | 0.5 | -0.1 | 0.77 | 3 |  | 10 | 8 | 0.6 | 0.7 | -0.1 | 0.96 | 3 |
| 1 | 15 | 0.7 | 0.3 | 0.4 | 0.95 | 2 |  | 10 | 11 | 0.6 | 0.4 | 0.2 | 0.53 | 2 |
| 2 | 13 | 0.6 | 0.7 | -0.1 | 0.97 | 1 |  | 10 | 12 | 0 | 0.2 | -0.2 | 0.40 | 5 |
| 2 | 16 | 0.2 | 0.2 | 0 | 0.95 | 4 |  | 10 | 16 | 0.1 | 0 | 0.1 | 0.16 | 1 |
| 2 | 17 | 0 | 0.3 | -0.3 | 1.04 | 3 |  | 11 | 4 | 0.8 | 0.8 | 0 | 0.60 | 1 |
| 2 | 18 | 0.8 | 0.8 | 0 | 1.20 | 2 |  | 11 | 7 | 0 | 0.4 | -0.4 | 0.45 | 4 |
| 3 | 1 | 0.3 | 0.6 | -0.3 | 0.80 | 4 |  | 11 | 15 | 0.6 | 0.5 | 0.1 | 0.70 | 5 |
| 3 | 8 | 0.5 | 0.2 | 0.3 | 0.74 | 5 |  | 11 | 16 | 0.1 | 0.1 | 0 | 0.18 | 2 |
| 3 | 12 | 0.1 | 0 | 0.1 | 0.80 | 2 |  | 12 | 6 | 0 | 0.4 | -0.4 | 1.26 | 3 |
| 3 | 15 | 1 | 1 | 0 | 0.97 | 3 |  | 12 | 10 | 0.2 | 0 | 0.2 | 1.14 | 2 |
| 4 | 9 | 0.1 | 0.7 | -0.6 | 1.12 | 5 |  | 12 | 15 | 0.6 | 0.3 | 0.3 | 1.55 | 1 |
| 4 | 11 | 0.7 | 0.9 | -0.2 | 0.87 | 2 |  | 12 | 18 | 0.7 | 0.7 | 0 | 1.67 | 5 |
| 4 | 14 | 0.3 | 0.2 | 0.1 | 0.41 | 1 |  | 13 | 2 | 0.7 | 0.6 | 0.1 | 1.02 | 4 |
| 4 | 19 | 0.4 | 0.5 | -0.1 | 0.78 | 4 |  | 13 | 7 | 0 | 0.3 | -0.3 | 0.58 | 5 |
| 5 | 14 | 0.2 | 0.4 | -0.2 | 0.91 | 2 |  | 13 | 9 | 0.2 | 0.2 | 0 | 0.67 | 1 |
| 5 | 17 | 0 | 0 | 0 | 0.78 | 3 |  | 13 | 11 | 0.5 | 0.3 | 0.2 | 0.65 | 3 |
| 5 | 18 | 0.7 | 0.7 | 0 | 1.25 | 5 |  | 15 | 1 | 0.3 | 0.7 | -0.4 | 0.68 | 3 |
| 5 | 19 | 0.4 | 0.4 | 0 | 0.96 | 4 |  | 15 | 3 | 1 | 1 | 0 | 1.72 | 4 |
| 6 | 1 | 0.2 | 0.4 | -0.2 | 1.31 | 3 |  | 15 | 8 | 0.5 | 0.4 | 0.1 | 0.73 | 2 |
| 6 | 9 | 0.2 | 0.2 | 0 | 1.26 | 2 |  | 15 | 17 | 0.1 | 0 | 0.1 | 0.70 | 1 |
| 6 | 18 | 0.4 | 0.1 | 0.3 | 1.57 | 4 |  | 17 | 1 | 0.6 | 0.6 | 0 | 1.04 | 5 |
| 6 | 19 | 0.9 | 0.9 | 0 | 2.06 | 5 |  | 17 | 5 | 0 | 0 | 0 | 0.69 | 4 |
| 7 | 1 | 0.9 | 0.8 | 0.1 | 2.32 | 5 |  | 17 | 12 | 0.6 | 0.4 | 0.2 | 1.08 | 1 |
| 7 | 12 | 0.4 | 0.6 | -0.2 | 0.68 | 1 |  | 17 | 15 | 0.2 | 0.1 | 0.1 | 0.97 | 2 |
| 7 | 14 | 0.5 | 0.3 | 0.2 | 1.36 | 4 |  | 18 | 2 | 0.8 | 0.8 | 0 | 0.95 | 3 |
| 7 | 16 | 0.1 | 0 | 0.1 | 1.01 | 3 |  | 18 | 6 | 0.1 | 0.4 | -0.3 | 0.83 | 1 |
| 8 | 6 | 0 | 0.4 | -0.4 | 0.51 | 1 |  | 18 | 11 | 0.4 | 0.3 | 0.1 | 0.63 | 2 |
| 8 | 7 | 0.2 | 0.2 | 0 | 0.91 | 4 |  | 18 | 15 | 0.2 | 0.2 | 0 | 0.59 | 4 |
| 8 | 10 | 0.7 | 0.6 | 0.1 | 0.93 | 2 |  | 19 | 4 | 0.5 | 0.4 | 0.1 | 0.77 | 5 |
| 8 | 13 | 0.6 | 0.4 | 0.2 | 1.35 | 5 |  | 19 | 6 | 0.9 | 0.9 | 0 | 0.90 | 3 |
| 9 | 3 | 0.5 | 0.4 | 0.1 | 1.23 | 1 |  | 19 | 11 | 0.4 | 0.4 | 0 | 0.90 | 1 |
| 9 | 4 | 0.6 | 0.2 | 0.4 | 1.14 | 2 |  | 19 | 14 | 0.2 | 0.5 | -0.3 | 0.54 | 4 |
